# Resistance to Androgen Deprivation Leads to Altered Metabolism in Human and Murine Prostate Cancer Cell and Tumor Models

**DOI:** 10.3390/metabo11030139

**Published:** 2021-02-26

**Authors:** Jinny Sun, Robert A. Bok, Justin DeLos Santos, Deepti Upadhyay, Romelyn DeLos Santos, Shubhangi Agarwal, Mark Van Criekinge, Daniel B. Vigneron, Rahul Aggarwal, Donna M. Peehl, John Kurhanewicz, Renuka Sriram

**Affiliations:** 1 Graduate Program in Bioengineering, University of California, Berkeley and University of California, San Francisco, CA 94143, USA; jinsuntea@gmail.com; 2 Department of Radiology and Biomedical Imaging, University of California, San Francisco, CA 94143, USA; robert.bok@ucsf.edu (R.A.B.); jmdeloss@gmail.com (J.D.S.); Deepti.Upadhyay@ucsf.edu (D.U.); Romelyn.DelosSantos@ucsf.edu (R.D.S.); Shubhangi.Agarwal@ucsf.edu (S.A.); Mark.VanCriekinge@ucsf.edu (M.V.C.); Dan.Vigneron@ucsf.edu (D.B.V.); donna.peehl@ucsf.edu (D.M.P.); 3 Divisions of Hematology & Oncology, University of California, San Francisco, CA 94143, USA; Rahul.Aggarwal@ucsf.edu

**Keywords:** metabolism, prostate cancer, castration-resistant, androgen-dependent, TRAMP, magnetic resonance, hyperpolarized [1-^13^C]pyruvate, lactate, glycolysis

## Abstract

Currently, no clinical methods reliably predict the development of castration-resistant prostate cancer (CRPC) that occurs almost universally in men undergoing androgen deprivation therapy. Hyperpolarized (HP) ^13^C magnetic resonance imaging (MRI) could potentially detect the incipient emergence of CRPC based on early metabolic changes. To characterize metabolic shifts occurring upon the transition from androgen-dependent to castration-resistant prostate cancer (PCa), the metabolism of [U-^13^C]glucose and [U-^13^C]glutamine was analyzed by nuclear magnetic resonance spectroscopy. Comparison of steady-state metabolite concentrations and fractional enrichment in androgen-dependent LNCaP cells and transgenic adenocarcinoma of the murine prostate (TRAMP) murine tumors versus castration-resistant PC-3 cells and treatment-driven CRPC TRAMP tumors demonstrated that CRPC was associated with upregulation of glycolysis, tricarboxylic acid metabolism of pyruvate; and glutamine, glutaminolysis, and glutathione synthesis. These findings were supported by ^13^C isotopomer modeling showing increased flux through pyruvate dehydrogenase (PDH) and anaplerosis; enzymatic assays showing increased lactate dehydrogenase, PDH and glutaminase activity; and oxygen consumption measurements demonstrating increased dependence on anaplerotic fuel sources for mitochondrial respiration in CRPC. Consistent with ex vivo metabolomic studies, HP [1-^13^C]pyruvate distinguished androgen-dependent PCa from CRPC in cell and tumor models based on significantly increased HP [1-^13^C]lactate.

## 1. Introduction

Androgen deprivation therapy (ADT) is the cornerstone of treatment for men with recurrent or metastatic prostate cancer (PCa). However, almost all patients eventually stop responding to ADT and develop castration-resistant prostate cancer (CRPC) [1]. Clinical diagnosis of CRPC is based on a significant increase in tumor burden or metastasis detected using computed tomography (CT) scans, bone scintigraphy or magnetic resonance imaging (MRI), and/or rising serum prostate-specific antigen (PSA) levels [2]. Currently, no reliable clinical or non-invasive imaging methods can specifically predict the development of CRPC, which is critical in guiding treatment decisions in men with advanced PCa. Early detection of emerging CRPC, prior to confirmation by prevailing clinical parameters, could expedite initiation of next-line therapies to slow or halt progression of disease.

Genomic analyses of PCa cell lines and patient tumors have shown that androgen receptor (AR) is still active in some subtypes of CRPC [3,4] despite castration levels of testosterone and that its DNA binding profile changes [5]. Expression of AR-regulated genes including those involved in glucose uptake and glycolysis, glutaminolysis, and anabolic metabolism have been shown to be altered in CRPC [6]. Furthermore, AR:DNA complex binding sites found only in the CRPC cistrome are enriched for MYC [5], which regulates glycolysis and glutaminolysis [7]. In subtypes of CRPC with little or no AR, other oncoproteins may induce similar metabolic changes [8]. Altogether, these genetic findings suggest that metabolism of glucose and glutamine is altered in CRPC.

Hyperpolarized (HP) ^13^C MRI is a powerful technology that can image metabolic fluxes through key pathways associated with cancer progression and therapeutic response [9]. Preclinical HP ^13^C MRI studies have shown that glycolytic activity, as measured by the rate of conversion of ^13^C pyruvate to ^13^C lactate (*k_PL_*) and the pyruvate-to-lactate ratio, increases as PCa become more aggressive [10,11,12]. Additionally, HP ^13^C MRI demonstrated an early reduction in *k_PL_* after ADT in a metastatic lesion of a patient with PCa, preceding clinical response to therapy [13]. While initial preclinical and patient studies have focused on using IND-approved HP [1-^13^C]pyruvate to investigate changes in glycolysis, clinical translation of HP probes that provide insight into other metabolic pathways, such as [2-^13^C]pyruvate and [5-^13^C]glutamine, is in progress.

Although HP ^13^C MRI has shown potential to monitor metabolic changes that occur during progression of PCa and in response to therapy, its relevance to detection of CRPC remains speculative pending further definition of the metabolic phenotype of CRPC. Here, human PCa cell lines representative of androgen-dependent PCa (LNCaP) and CRPC (PC-3) were compared to identify metabolic pathways related to the castration-resistant phenotype. To more closely mimic the in vivo progression from androgen dependence to CRPC, the transgenic adenocarcinoma of the mouse prostate (TRAMP) model was also studied [14]. Similar to human PCa, TRAMP tumors are initially androgen-dependent and progress to CRPC upon resistance to orchiectomy, which is equivalent to chemically-induced ADT [15,16]. The metabolism of [U-^13^C]glucose and [U-^13^C]glutamine in these models was evaluated by 1D and 2D high-field nuclear magnetic resonance (NMR) spectroscopy. Measurement of steady-state levels and fractional enrichment of metabolites accompanied by ^13^C isotopomer modeling, enzymatic assays, and determination of rates of oxygen consumption demonstrated an upregulation of glycolysis, TCA cycle, glutaminolysis and glutathione synthesis in CRPC. Finally, the ability of HP [1-^13^C]pyruvate NMR spectroscopy to distinguish PC-3 from LNCaP cells and androgen-dependent versus CRPC TRAMP tumors due to upregulated glycolysis was demonstrated.

## 2. Results

### 2.1. Differences in Steady State Levels of Metabolites between Androgen-Dependent and Castration-Resistant PCa

**Figure 1:**
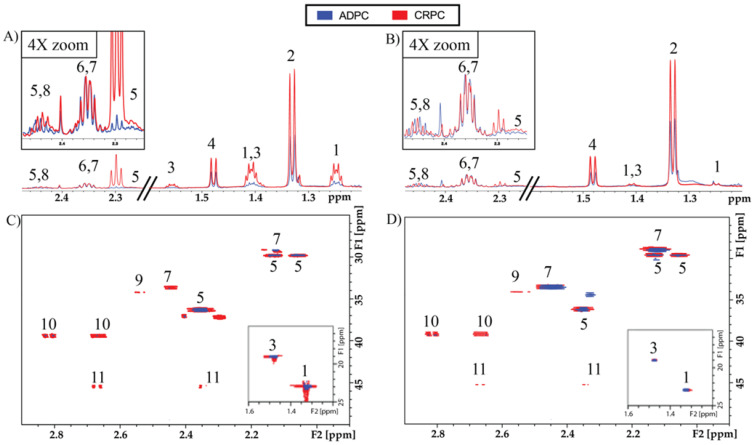
High-resolution NMR spectra of tumor extracts. Representative 1D ^1^H spectra with water presaturation of ADPC (blue) and CRPC (red) TRAMP tumors labeled with (**A**) [U-^13^C]glucose, and (**B**) [U-^13^C]glutamine. Zoomed region indicates glutamate C4 region with vertical scale increased by 4-fold. High resolution 2D ^1^H-^13^C HSQC of ADPC and CRPC TRAMP tumors labeled with (**C**) [U-^13^C]glucose, and (**D**) [U-^13^C]glutamine. Metabolite peaks are labeled as follows: (1) ^13^C-Lactate, (2) Lactate, (3) ^13^C-Alanine, (4) Alanine, (5) ^13^C-Glutamate, (6) Glutamate, (7) ^13^C-Glutamine, (8) Glutamine, (9) ^13^C-Glutathione, (10) ^13^C-Aspartate, (11) ^13^C-Malate.

**Figure 2:**
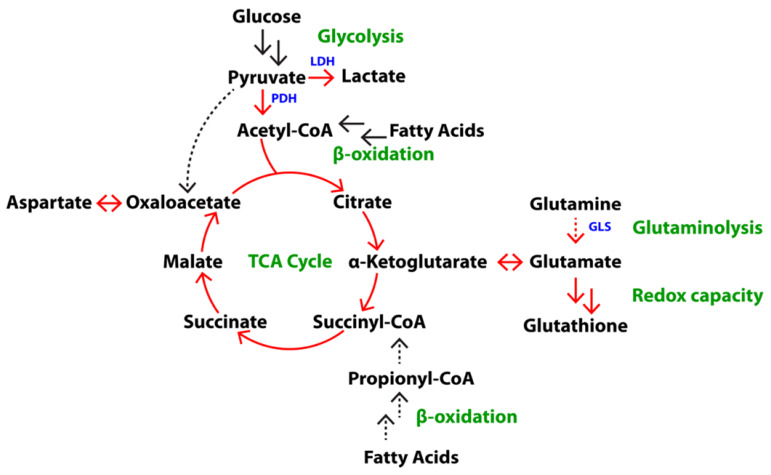
Overview of metabolic pathways associated with glucose and glutamine metabolism. Key metabolic pathways (green) and respective metabolites (black) and enzymes (blue) are shown. Red arrows indicate pathways that are particularly relevant to distinguishing CRPC from ADPC, doubled arrows represent simplified multistep processes, and dashed lines indicate anaplerotic pathways of the TCA cycle.

To assess utilization of glucose and glutamine in androgen-dependent PCa (ADPC) versus CRPC, LNCaP and PC-3 cells were labeled with [U-^13^C]glucose or [U-^13^C]glutamine in vitro, and ADPC and CRPC TRAMP tumors were labeled in vivo in mice infused with [U-^13^C]glucose or [U-^13^C]glutamine. Soluble metabolites extracted from cells and tumors were analyzed by high-field NMR spectroscopy. Representative 1D ^1^H spectra with water presaturation clearly highlight the differences in the metabolic flux of glucose and glutamine between the ADPC versus CRPC cell lines (Figure S1A,B) and TRAMP tumors (Figure 1A,B). Total and ^13^C-labeled metabolite concentrations were quantified using ^13^C-decoupled ^1^H spectroscopy (Figure S2) and 2D ^1^H-^13^C HSQC (Figure 1C,D and Figure S1C,D), respectively. Steady-state concentrations of metabolites in each of the cell lines and TRAMP tumors are listed in Supplementary Table S1. Levels of aspartate, glutamate, lactate, myo-inositol, phosphocholine, glycerophosphocholine, and total choline were significantly higher in castration-resistant PC-3 cells compared to androgen-dependent LNCaP cells, while levels of citrate, glucose, creatine, and creatine phosphate were significantly lower in PC-3 compared to LNCaP cells. Similarly, levels of lactate were significantly higher, and citrate and creatine were significantly lower, in CRPC compared to ADPC TRAMP tumors. A schematic of the metabolic pathways involving several of these metabolites is shown in Figure 2. Altogether, the results demonstrate the components of glycolysis (glucose, lactate), the TCA cycle (aspartate, citrate, glutamate), glutaminolysis (glutamate) and redox capacity (glutathione) are altered in CRPC compared to ADPC.

### 2.2. Elevated Glycolysis in CRPC versus ADPC

**Figure 3:**
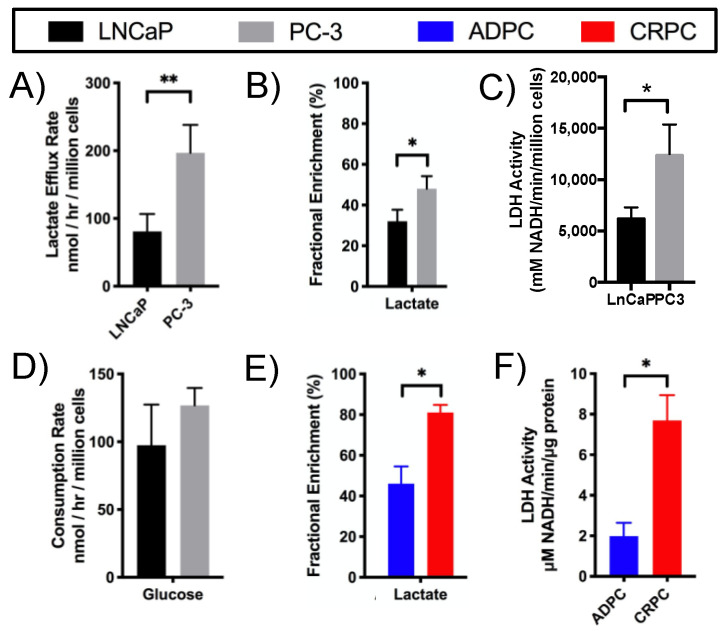
CRPC cells and tumors exhibit elevated glycolytic activity. (**A**) Lactate efflux rate and (**B**) steady-state fractional enrichment of lactate were quantified in LNCaP and PC-3 cells labeled with [U-^13^C]glucose (*n* = 3). (**C**) Enzymatic activity of LDH in LNCaP and PC-3 cells was determined by biochemical assay of protein lysates (*n* = 3). (**D**) Steady-state consumption rates of [U-^13^C]glucose by LNCaP and PC-3 cells (*n* = 3). (**E**) Fractional enrichment of lactate and (**F**) LDH activity were determined in ADPC and CRPC TRAMP tumors labeled with [U-^13^C]glucose (*n* = 3). (* *p* < 0.05, ** *p* < 0.01)

In addition to higher steady-state levels of lactate in PC-3 compared to LNCaP cells (Supplementary Table S1), the rate of lactate efflux from cells into the culture medium was also significantly higher for PC-3 compared to LNCaP cells (Figure 3A). Quantification of the fractional enrichment (FE) of lactate in ^13^C-glucose labeling studies showed that PC-3 cells had significantly increased lactate FE compared to LNCaP cells (Figure 3B), indicating increased glucose flux through glycolysis (Figure 2). This finding was supported by the significantly elevated enzymatic activity of lactate dehydrogenase (LDH), which converts pyruvate to lactate (Figure 2), in PC-3 compared to LNCaP cells (Figure 3C). The consumption rate of glucose, however, did not differ between the cell lines (Figure 3D). Together, these data indicate that glucose flux through glycolysis is upregulated in PC-3 relative to LNCaP cells.

The glycolytic alterations occurring in cell lines in vitro were recapitulated in vivo in the TRAMP model mimicking the development of CRPC in patients. In [U-^13^C]glucose-labeling studies, FE of lactate was significantly higher in CRPC compared to ADPC TRAMP tumors (Figure 3E), indicating increased flux of glucose through glycolysis as seen in PC-3 compared to LNCaP cells. Moreover, similar to the cell lines, LDH activity was higher in CRPC compared to ADPC TRAMP tumors (Figure 3F). In all, higher levels of steady-state lactate, increased FE of lactate in ^13^C-glucose labeling studies, and elevated activity of LDH indicate that glycolysis is upregulated in human and murine models of CRPC compared to their androgen-dependent counterparts.

### 2.3. Increased Utilization of Pyruvate in the TCA Cycle in CRPC versus ADPC

The TCA cycle is a central metabolic pathway that supports aerobic respiration, macromolecule synthesis, and redox balance. As shown in Figure 2, several different metabolites can fuel the TCA cycle, including pyruvate, glutamine, and fatty acids. Preferences in substrate utilization as well as the overall activity of the TCA cycle can be characterized by measuring the FE of aspartate and glutamate, both of which are in fast exchange with low-abundance intermediates of the TCA cycle [17]. In addition, the intermediates of the TCA cycle can be used to fuel macromolecule synthesis of carbohydrates, lipids, and proteins. The role of the TCA cycle in prostate metabolism is particularly interesting, as the healthy prostate gland is responsible for producing and secreting large amounts of citrate. This results in a truncated TCA cycle [18]. In PCa, citrate is oxidized to support energy production and the TCA cycle is restored. However, the metabolic activity of the TCA cycle in CRPC is unknown.

**Figure 4:**
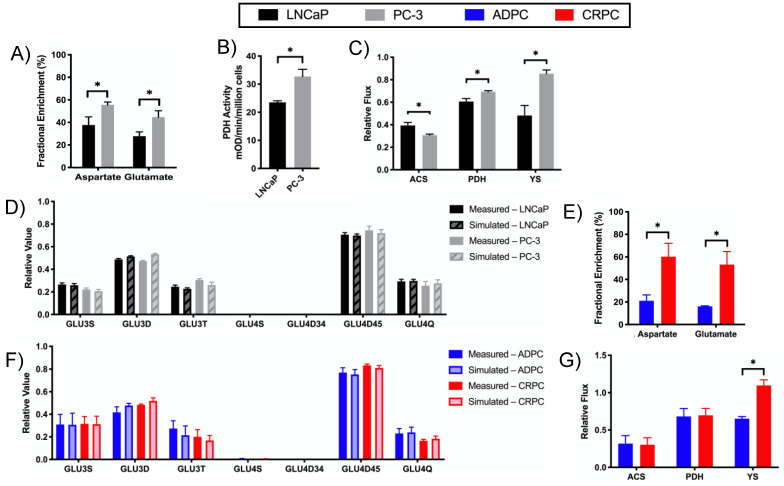
Pyruvate oxidation is increased in CRPC to fuel the TCA cycle. Flux of pyruvate through the TCA cycle was assessed in LNCaP and PC-3 cells by measuring (**A**) the fractional enrichment of aspartate and glutamate in cells labeled with [U-^13^C]glucose and (**B**) enzymatic activity of PDH. (**C**) Calculated values for the relative flux of unlabeled acetate and other fatty acids into the TCA cycle (ACS), relative ^13^C-glucose flux into the TCA cycle (PDH), and the anaplerotic contribution relative to citrate synthase (YS) using TCACALC. (**D**) ^13^C isotopomer modeling of [U-^13^C]glucose-labeled LNCaP and PC-3 cells using TCACALC showed measured and simulated relative ^13^C isotopomer areas of the glutamate C3 and C4 multiplets. Flux of pyruvate through the TCA cycle was determined in ADPC and CRPC TRAMP tumors by measuring (**E**) fractional enrichment of aspartate and glutamate of tumors labeled with [U-^13^C]glucose. ^13^C isotopomer modeling of [U-^13^C]glucose-labeled ADPC and CRPC TRAMP tumors using TCACALC showed (**F**) measured and simulated relative ^13^C isotopomer areas of the glutamate C3 and C4 multiplets and (**G**) calculated values for the relative flux of ACS, PDH, and YS. (* *p* < 0.05).

The differential metabolism of the TCA cycle is demonstrated by the [U-^13^C]glucose-labeling results shown in Figure 4A, which revealed that PC-3 compared to LNCaP cells had significantly increased FE of aspartate and glutamate, suggesting increased glucose utilization through the TCA cycle in PC-3 cells (Figure 2). The enzymatic activity of pyruvate dehydrogenase (PDH), which converts pyruvate into acetyl-CoA (Figure 2), was also higher in PC-3 than LNCaP cells (Figure 4B). To corroborate these findings, ^13^C isotopomer modeling using TCACALC was performed using the relative isotopomer ratios of glutamate derived from [U-^13^C]glucose in LNCaP and PC-3 cells. ^13^C isotopomer modeling of [U-^13^C]glucose-labeled cell extracts indicated significantly upregulated relative PDH flux in PC-3 cells (Figure 4C). A good fit of the model was observed as reflected by the simulated isotopomer ratios of glutamate closely matching experimental values (Figure 4D). Furthermore, over 95% of the pyruvate is uniformly labeled, indicating that the majority of pyruvate entering the TCA cycle results from [U-^13^C]glucose. These studies demonstrated that PC-3 cells had more pyruvate entering the TCA cycle via pyruvate oxidation by PDH than other sources such as acetate (via increased ACS activity) and other sources resulting from β-oxidation of fatty acid (Figure 2). Together, these results illustrate that fractional glucose flux through the TCA cycle is higher in PC-3 cells, whereas in LNCaP cells, although the uptake of glucose is similar, the utilization of glucose for the TCA cycle is lower.

Similar to the castration-resistant versus androgen-dependent cell lines, CRPC TRAMP tumors exhibited increased FE of glutamate and aspartate compared to ADPC TRAMP tumors after labeling with [U-^13^C]glucose, suggesting increased pyruvate flux through TCA metabolism (Figure 4E). Altogether, increased utilization of glucose in the TCA cycle was characteristic of both human and murine models of CRPC compared to ADPC. Interestingly, ^13^C isotopomer modeling of [U-^13^C]glucose-labeled ADPC and CRPC TRAMP tumors (Figure 4F,G) revealed no significant difference in relative PDH flux, which is also what we observed in biochemical assay (data not shown). This could indicate pyruvate cycling (via malic enzyme) that is not captured in this modeling. We were, however, not able to robustly quantify the C2 isotopomer pattern (presence of C12 doublets [19,20]) of the lactate peak to confirm this due to low SNR.

### 2.4. Glutamine Utilization in the TCA Cycle and Glutaminolysis in CRPC versus ADPC

**Figure 5:**
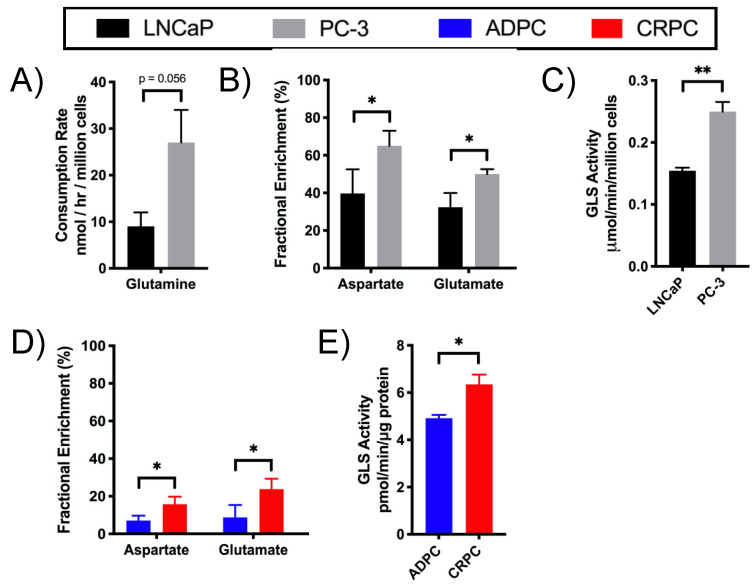
Glutamine metabolism through glutaminolysis and the TCA cycle is enhanced in CRPC. Glutamine metabolism was assessed in LNCaP and PC-3 cells based on (**A**) consumption rate of [U-^13^C]glutamine, (**B**) the fractional enrichment of aspartate and glutamate of cells labeled with [U-^13^C]glutamine, and (**C**) enzymatic activity of GLS. Flux through glutaminolysis and the TCA cycle was determined in ADPC and CRPC TRAMP tumors based on (**D**) the fractional enrichment of aspartate and glutamate of tumors labeled with [U-^13^C]glutamine and (**E**) enzymatic activity of GLS. (* *p* < 0.05, ** *p* < 0.01).

^13^C isotopomer modeling indicated that PC-3 cells had significantly higher relative anaplerotic flux (YS) compared to LNCaP cells (Figure 4C). To determine the anaplerotic fuel source, glutamine metabolism and its relative anaplerosis to the TCA cycle were assessed. As described above (Supplementary Table S1), PC-3 compared to LNCaP cells had significantly higher concentrations of steady-state glutamate, which could be generated directly from glutamine (Figure 2). Furthermore, [U-^13^C]glutamine-labeling studies showed that PC-3 cells consumed glutamine at a significantly higher rate than LNCaP cells (Figure 5A). PC-3 cells also exhibited increased glutamine flux through glutaminolysis and the TCA cycle based on the significantly elevated FE of aspartate and glutamate in PC-3 compared to LNCaP cells (Figure 5B). Correspondingly, PC-3 cells also had significantly increased activity of glutaminase (GLS), which converts glutamine to glutamate, compared to LNCaP cells (Figure 5C), in accord with the significantly increased activity through glutaminolysis observed after [U-^13^C]glutamine labeling. In sum, these results indicate that glutaminolysis and its subsequent anaplerosis are upregulated in PC-3 compared to LNCaP cells.

Glutaminolysis and glutamine anaplerosis into the TCA cycle were then assessed in the TRAMP model. While the steady-state concentrations of aspartate and glutamate were not significantly different between ADPC and CRPC TRAMP tumors (Supplementary Table S1), [U-^13^C]glutamine-labeling studies revealed that CRPC TRAMP tumors had significantly increased FE of aspartate and glutamate compared to ADPC tumors, demonstrating increased flux through glutaminolysis and increased anaplerosis into the TCA cycle (Figure 5D). The upregulation of glutaminolysis in CRPC tumors is further supported by significantly higher activity of GLS in CRPC versus ADPC TRAMP tumors (Figure 5E). Interestingly, the low fractional enrichment in TRAMP tumors shows that they are not as glutamine-reliant as LNCaP and PC-3 cells. Altogether, these results indicate that upregulation of glutaminolysis and glutamate anaplerosis into the TCA cycle are characteristic of castration-resistant versus androgen-dependent pCa.

### 2.5. Differential Substrate Utilization into Oxidative Phosphorylation (OXPHOS) by LNCaP and PC-3 Cells

**Figure 6:**
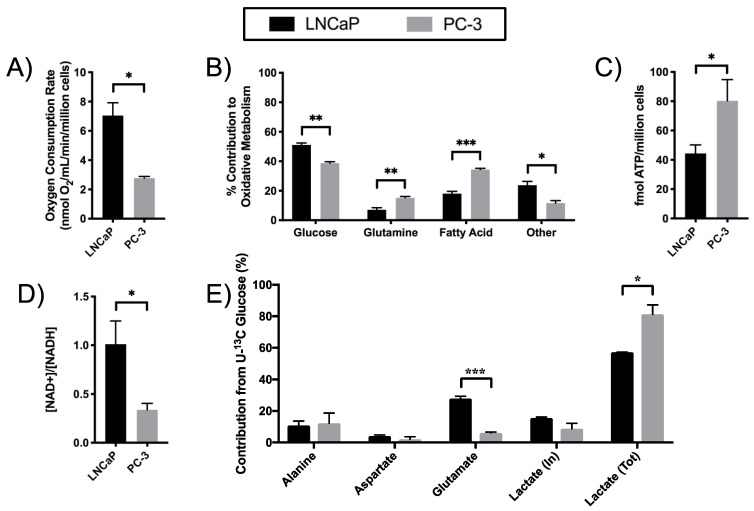
Cellular respiration and bioenergentics are altered in CRPC compared to ADPC. Oxygen consumption rate and relative substrate utilization for TCA metabolism of LNCaP and PC-3 cells. (**A**) Oxygen consumption rate of LNCaP and PC-3 cells was measured using a Clark-type O_2_ electrode. (**B**) The relative contributions of glucose, long-chain fatty acids, and glutamine to the TCA cycle in PCa cells was assessed using the Seahorse XFe24 Mito Fuel Flex assay. Cellular bioenergetics of LNCaP and PC-3 cells was assessed based on (**C**) ATP content and (**D**) intracellular NAD^+^/NADH. (**E**) Relative mass contribution of ^13^C-glucose to downstream metabolites. Lactate (in) refers to intracellular and (tot) refers to total of the intracellular and extracellular pools of ^13^C labeled lactate. (* *p* < 0.05, ** *p* < 0.01, *** *p* < 0.001).

To better understand the relative utilization of glucose, long-chain fatty acids, and glutamine in the TCA cycle by ADPC versus CRPC, the oxygen consumption rate (OCR) was measured using the Oxygraph+ and substrate preferences were assessed using the Seahorse XFe24 Extracellular Flux Analyzer. LNCaP cells displayed higher basal OCR compared to PC-3 cells (Figure 6A), suggesting that LNCaP cells could have higher oxidative phosphorylation of glucose and beta-oxidation compared to PC-3 cells. Using these oxygen consumption values, the total TCA flux of the two cells was calculated [21] to be 3.1 ± 0.4 and 1 ± 0.1 nmol/min/million cells in LNCaP and PC-3 cells, respectively. From this, the absolute PDH flux was determined to be 1.8 ± 0.1 and 0.7 ± 0.1 nmol/min/million cells in LNCaP and PC-3. However, the relative contribution of glucose to the TCA flux (ratio of PDH flux to total TCA flux) remains significantly higher (*p* = 0.038) in PC-3 (0.67 ± 0.01) cells compared to LNCaP (0.58 ± 0.04) cells as observed in Section 2.3. Similarly, while the absolute anaplerotic flux is higher in the LNCaP cells (1.5 ± 0.3 compared to 0.72 ± 0.16 nmol/min/million cells in PC-3), the relative contribution of the anaplerotic sources to TCA flux is 1.5 times higher in PC-3 cells compared to LNCaP (0.74 ± 0.17 and 0.48 ± 0.13 respectively). This is in line with the observation of higher contribution of OXPHOS of glucose in LNCaP cells (Figure 6B), resulting in increased glucose conversion to glutamate (Figure 6E). Furthermore, the increased relative ACS flux from TCACALC modeling data (Figure 4C) highlights the contribution of other substrates (such as acetate as a source of acetyl CoA) to the TCA activity in LNCaP cells. While glucose was the primary fuel source for OXPHOS in both cell lines when measured using the Seahorse Mito Fuel Flex kit, PC-3 cells relied more on glutamine and long-chain fatty acids as fuel sources compared to LNCaP cells. Alternatively, LNCaP cells relied more on short and medium chain fatty acids (that serve as precursors to the anaplerotic substrate propionyl CoA), amino acids that enter through oxaloacetate (e.g., aspartate and asparagine), and branched-chain amino acids that enter through succinyl-CoA (e.g., leucine, isoleucine, and valine) [22].

Cellular bioenergetics were also assessed to understand the differential upregulation of glycolysis and the TCA cycle to meet the energy needs of LNCaP and PC-3 cells. The total ATP content was significantly higher in PC-3 than LNCaP cells (Figure 6C), suggesting that PC-3 cells have higher energy capacity. Furthermore, PC-3 cells had a significantly lower NAD^+^/NADH ratio compared to LNCaP cells (Figure 6D), which is consistent with the significantly upregulated glycolysis in PC-3 cells. This is further supported by the mass balance analysis of ^13^C-glucose-labeled metabolites, which demonstrated that PC-3 cells utilized the majority of glucose consumed to produce lactate (Figure 6E).

### 2.6. Increased Redox Capacity in CRPC versus ADPC

**Figure 7:**
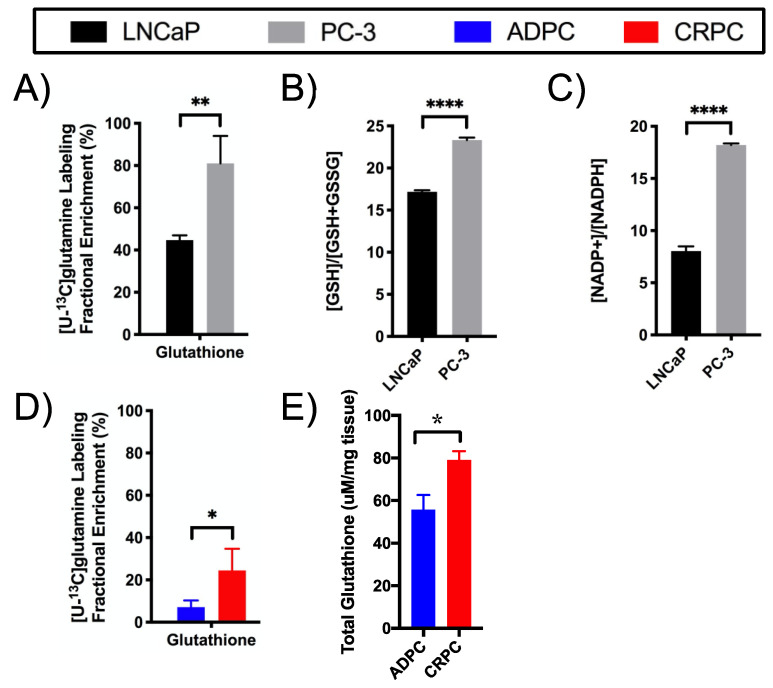
Glutathione redox capacity is upregulated in CRPC. Glutathione redox capacity was assessed in LNCaP and PC-3 cells based on (**A**) fractional enrichment of glutathione in cells labeled with [U-^13^C]glutamine, (**B**) ratio of reduced glutathione (GSH) relative to total glutathione (GSH + GSSG), and (**C**) intracellular NADP^+^/NADPH. Glutathione redox balance was assessed in ADPC and CRPC TRAMP tumors based on (**D**) fractional enrichment of glutathione in tumors labeled with [U-^13^C]glutamine and (**E**) total glutathione (* *p* < 0.05, ** *p* < 0.01, **** *p* < 0.0001).

The significantly higher level of steady-state glutathione in PC-3 compared to LNCaP cells (Supplementary Table S1) suggests a difference in redox capacity between the two types of cells. Furthermore, [U-^13^C]glutamine-labeling studies revealed that PC-3 cells had significantly increased FE of glutathione, quantified using 2D ^1^H-^1^H TOCSY (Supplementary Figure S1E), compared to LNCaP cells (Figure 7A). This is a true reflection of an increase in glutathione synthesis and not simply a consequence of increased glutamate FE in PC-3 as reflected by the ratio of glutathione to glutamate FE which was 80 ± 0 and 114 ± 0.1 in LNCaP and PC-3 cells, suggesting increased glutamine flux through glutathione synthesis to support redox balance (Figure 2). This correlates with the significantly increased ratio of reduced glutathione relative to total glutathione (Figure 7B) and increased NADP^+^/NADPH ratio (Figure 7C) in PC-3 compared to LNCaP cells. Taken together, these results suggest that PC-3 cells have increased glutathione redox capacity compared to LNCaP cells.

[U-^13^C]glutamine-labeling studies also showed that CRPC TRAMP tumors had significantly elevated FE of glutathione compared to ADPC tumors (Figure 6D), suggesting increased glutathione synthesis to support redox balance. The biochemical assay revealed a significantly higher total glutathione pool in CRPC relative to ADPC tumors (Figure 6E). Overall, these results suggest that CRPC has higher glutathione redox potential compared to ADPC.

### 2.7. Elevated Glycolysis Observed in CRPC vs. ADPC Using HP [1-^13^C]pyruvate

**Figure 8:**
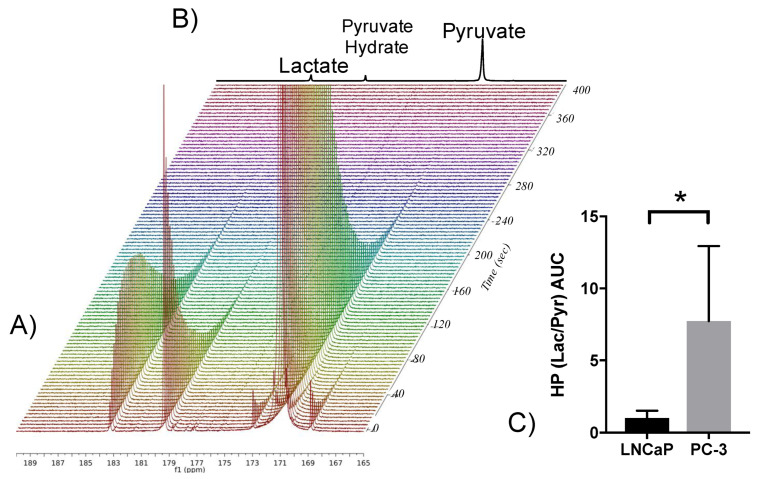
Increased glycolytic activity of CRPC is measured in real-time using HP [1-^13^C]pyruvate. (**A**) Representative dynamic spectra of hyperpolarized [1-^13^C] pyruvate being converted to [1-13C] lactate in PC-3 cells. (**B**) summed HP ^13^C spectra show the lactate signal that arises from [1-^13^C]pyruvate. (**C**) Mean HP (Lac/Pyr) area under the curve (AUC) (* *p* < 0.05, *n* = 4).

The metabolic differences identified between ADPC and CRPC led us to assess the ability of HP [1-^13^C]pyruvate NMR spectroscopy to measure increased glycolysis in PC-3 over LNCaP cells. Dynamic 1D ^13^C NMR spectral data were acquired from slurries of live cells (Figure 8A,B) and peak areas were integrated over time. PC-3 cells had a significantly higher pyruvate-to-lactate conversion AUC, than LNCaP cells, consistent with the upregulation of glycolysis as described by the metabolomics findings (Figure 8C). This demonstrates that HP [1-^13^C]pyruvate can be used to noninvasively assess glycolytic capacity.

**Figure 9:**
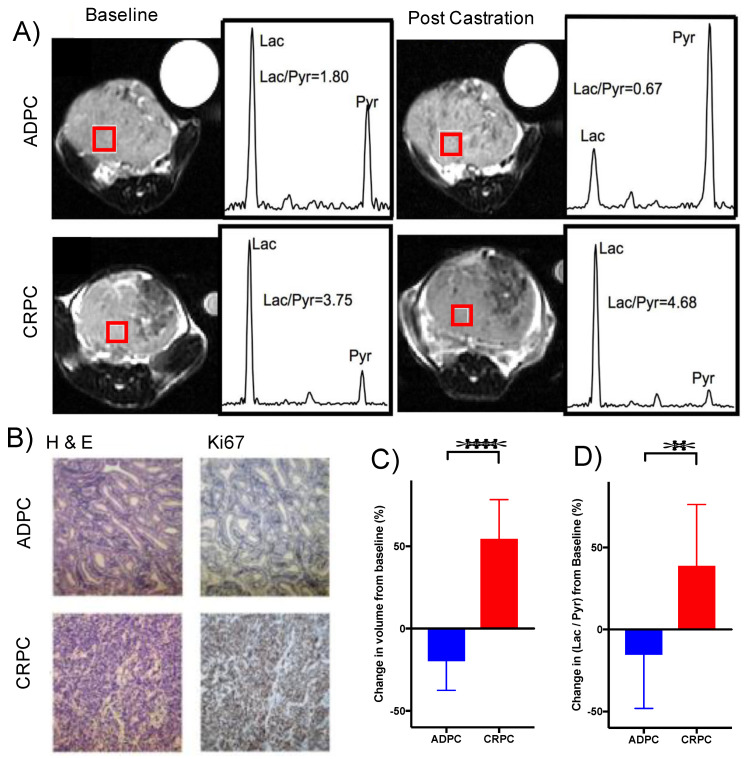
In vivo metabolism of HP [1-^13^C]pyruvate in a treatment-driven TRAMP model. (**A**) Representative T2-weighted proton images of ADPC (top row) and CRPC (bottom row) TRAMP tumors at baseline (left column) and 5 days post-castration (right column). The spectrum from a voxel (red square) is shown on the right of each image of the HP lactate (Lac) and pyruvate (Pyr) and the calculated ratio (Lac/Pyr). (**B**) Representative images of immunohistochemical staining of ADPC (top row) and CRPC (bottom row) of hematoxylin and eosin (H&E) and Ki67 (indicator of proliferation). (**C**) Change in volume and (**D**) HP Lac/Pyr of TRAMP tumors from baseline to post-orchiectomy in ADPC (*n* = 11) and CRPC (*n* = 7) mice. (** *p* < 0.01, **** *p* < 0.0001)

Metabolism of HP [1-^13^C]pyruvate was assessed in vivo in the orchiectomy-driven TRAMP model of CRPC using 3D chemical shift imaging (CSI) (Figure 9A). There was no significant difference in the baseline (pre-orchiectomy) volumes between the tumors that exhibited sensitivity to androgen deprivation versus those that exhibited castration-resistance post-orchiectomy (Supplementary Figure S3A). Similarly, no significant difference in HP Lac/Pyr ratio was observed between ADPC and CRPC tumors prior to orchiectomy (Supplementary Figure S3B). Representative images and spectrum from both the ADPC and CRPC tumors at baseline and subsequent follow-up are shown in Figure 9A. On average, the mice in the ADPC group survived (46 ± 10 days) twice as long as those in the CRPC group (20 ± 7 days). Immunohistochemical staining of Ki67 (Figure 9B) clearly shows the increased proliferation in CRPC tumors (greater than 90% compared to ~1% staining in ADPC tumors, Supplementary Figure S3C). There was a mean reduction in tumor volume observed in the ADPC group compared to a significant doubling of tumor volume in the CRPC group after castration (Figure 9C). There was also a significant reduction in glycolysis as evidenced by the reduction in the mean HP Lac/Pyr ratio in ADPC relative to the CRPC tumors (Figure 9D). This is in accord with our ex vivo metabolic findings using stable isotope tracers as well as the changes observed between the LNCaP and PC-3 cells (Figure 8C) and provides support for using the HP Lac/Pyr ratio as a marker of therapeutic resistance to ADT.

## 3. Discussion

In this study, metabolic changes associated with the development of CRPC were characterized in human PCa cell lines and in the TRAMP murine model of progression of androgen dependence to castration-resistance. Steady-state metabolite concentrations and FE were measured in [U-^13^C]glucose- and [U-^13^C]glutamine-labeled cells and tumors using high-resolution 1D and 2D NMR techniques, which revealed that CRPC exhibited increased flux through glycolysis, TCA metabolism of pyruvate and glutamine, glutaminolysis, and glutathione synthesis in both the human and murine models. This suggests that CRPC tumors have increased bioenergetic and biosynthetic demands to support castration-resistant growth.

Our finding that PC-3 cells had increased lactate production and export compared to LNCaP cells is consistent with previous findings [23]. We observed that LNCaP and PC-3 cells have similarly high rates of glucose consumption, in accordance with other studies that showed upregulation of glycolysis despite no significant difference in glucose consumption in castration-resistant cell and xenograft models [23,24,25,26].

Interestingly, PC-3 cells had lower OCR than LNCaP cells despite having higher PDH activity. This matches published studies which concluded that LNCaP cells rely mainly on OXPHOS for ATP generation while PC-3 cells rely mainly on glycolysis [27,28]. Previous studies have shown that AR signaling promotes a truncated TCA cycle by stimulating anabolism of glucose to citrate, resulting in significantly higher citrate levels but no change in succinate levels [6]. This is supported by the significantly higher steady-state citrate concentration but no significant difference in succinate in androgen-dependent LNCaP compared to castration-resistant PC-3 as shown in Supplementary Table S1.

One key metabolic characteristic of CRPC is glutamine addiction. Increased glutamine consumption and subsequent utilization in glutaminolysis, glutamine anaplerosis into the TCA cycle, and glutathione synthesis observed in our study of CRPC cells and tumors has previously been reported [29]. In addition to supporting bioenergetic and biosynthetic needs, increased glutaminolysis is required for activation of the mTORC1 signaling pathway, which upregulates metabolism of glucose, glutamine and other amino acids, and lipids [30]. Interestingly, increased glutaminolysis also supported upregulation of glutathione-dependent redox pathways in both castration-resistant PC-3 cells and the treatment-emergent CRPC TRAMP model. Several studies have shown that CRPC has higher redox capacity based on increased expression of glutathione-related antioxidant genes such as glutathione peroxidase (GPX2) and glutathione synthase (GSS) [31,32]. This may be a critical step in the development of CRPC as glutathione prevents apoptosis by neutralizing free radicals and reactive oxygen species that would otherwise cause oxidative damage to DNA, proteins, and lipids [33].

To our knowledge, we provide the first analysis of metabolic changes that occur as the TRAMP murine model of PCa progresses from androgen-dependence to CRPC after ADT. The TRAMP model is considered to be highly representative of human PCa [34]. We previously described the role of lactate metabolism in the initial stages of development of cancer in the prostates of TRAMP mice. Dual agent HP MRI with ^13^C-pyruvate and ^13^C-urea demonstrated an increased Warburg effect, with associated increase in activity of LDH, in high-grade tumors compared to low-grade tumors and the normal prostate [35]. In a triple-transgenic mouse model of PCa, knockdown of LDHA slowed primary tumor growth and development of metastases, implicating LDH activity and lactate production in progression of PCa [35].

PC-3 cells and CRPC TRAMP tumors are representative of an amphicrine subtype of CRPC that lacks expression of AR but is not double-negative, small cell neuroendocrine cancer (AR^−^/NE^−^) [36]. Nevertheless, many metabolic features of these castration-resistant models are similar to CRPC that retains expression of AR and/or AR splice variants. In the absence of AR, other factors may induce a similar metabolic transition. For instance, Connelly et al. reported that Foxa2 activated transcription of a subset of AR target genes in AR-negative CRPC TRAMP tumors [37]. Bluemn et al. found that elevated activity of FGF and MAPK pathways bypassed dependence on AR in AR-negative PCa [8]. Nevertheless, other studies suggest metabolic differences in AR-driven CRPC versus AR-negative CRPC. Bader et al. reported findings from an isotopomeric metabolic flux analysis that showed that AR-positive PCa had elevated expression of mitochondrial pyruvate carrier (MPC2), which facilitates import of cytosolic glucose-derived pyruvate into the mitochondrial matrix for subsequent metabolism into the TCA cycle, compared to AR-negative PCa [38]. Furthermore, the authors showed that AR-negative PCa models that lack MPC expression had increased glutamine uptake to support glutamine anaplerosis into the TCA cycle, while androgen-dependent PCa relied on glucose-derived pyruvate rather than glutamine as the major carbon source of the TCA cycle.

Finally, we demonstrated that PC-3 cells converted HP ^13^C-pyruvate to lactate at a higher rate than LNCaP cells, consistent with results from Zacharias et al. who examined PC-3 xenografts using HP ^13^C-pyruvate MRI [39]. However, recent work by van Heijster et al. [40] did not reveal a significant difference using the same cell lines, which could be attributed to the much higher concentration of hyperpolarized pyruvate used in our study. Further, we have shown in an inducible model of castration resistance that ADPC tumors are much less glycolytic than CRPC tumors and can be monitored noninvasively using HP [1-^13^C]pyruvate based on a significant increase in the HP ^13^C -lactate/^13^C -pyruvate ratio. HP ^13^C-pyruvate MRI is FDA-approved as an IND for PCa and undergoing clinical trials in a number of centers around the world for diverse applications, including predicting aggressiveness of primary PCa (NCT02526368) and response to therapy (NCT02911467) [41]. In a recent patient study, HP ^13^C-pyruvate MRI showed reduction in conversion of pyruvate to lactate as an early response to ADT that associated with therapeutic efficacy [13]. Ongoing pre-clinical studies also indicate that other HP probes that are under development, including [2-^13^C]pyruvate to assess TCA metabolism, [5-^13^C]glutamine to assess glutaminolysis, and [1-^13^C]dehydroascorbate to assess glutathione redox potential, could be developed as noninvasive imaging biomarkers of CRPC.

In summary, our findings using human and murine models are consistent with the hypothesis that castration-resistant growth alters several metabolic pathways, including glycolysis, glutaminolysis, TCA metabolism, and glutathione redox capacity. These pathways may provide biomarkers to predict the development of clinical CRPC or candidate drug targets for the treatment of CRPC in patients.

## 4. Materials and Methods

### 4.1. Cell Culture, ^13^C-Labeling, and Extraction of Metabolites

LNCaP (clone FGC, ATCC CRL-1740) and PC-3 (ATCC CRL-1435) cells were obtained from American Type Culture Collection (ATCC) and grown to 70–80% confluency in a 37 °C incubator with 95% air/5% CO_2_. For metabolic profiling, cells were cultured in RPMI-1640 medium (LNCaP) or Ham’s F-12K medium (PC-3) supplemented with 10% fetal bovine serum (FBS) and 1% penicillin–streptomycin for 6 h. For ^13^C-glucose labeling, media were replaced with glucose- and glutamine-free RPMI-1640 supplemented with 25 mM [U-^13^C]glucose, 10 mM glutamine, 10% FBS, and 1% penicillin–streptomycin for 6 h. For ^13^C-glutamine labeling, media were replaced with glucose- and glutamine-free RPMI-1640 supplemented with 10 mM [U-^13^C]glutamine, 25 mM glucose, 10% FBS, and 1% penicillin–streptomycin for 24 h. Culture media were collected at baseline and after labeling to quantify glucose consumption rate, glutamine consumption rate, and lactate export rate. Prior to extraction of metabolites, cells were rinsed with cold phosphate-buffered saline (PBS). Cellular metabolism was quenched by direct addition of cold methanol, and intracellular metabolites were extracted using cold 1:1:1 methanol:water:chloroform [42]. The aqueous fraction was isolated, lyophilized, and resuspended in 600 μL of D_2_O with TSP for NMR analysis.

### 4.2. Generation of Androgen-Dependent and CRPC TRAMP Tumors, ^13^C-Labeling, and Extraction of Metabolites

Adult TRAMP mice were obtained from Roswell Park Cancer Institute. Mice with a solid tumor mass between 0.1 and 1 cc underwent orchiectomy. Tumor volume was monitored by T_2_-weighted spin echo MRI and <25% increase ~5 days (±2 days) post-orchiectomy was defined as androgen-dependent prostate cancer (ADPC), while >35% increase was defined as CRPC. Tumor volume changes are depicted in Supplementary Figure S4 and are similar to the cohort of mice that underwent hyperpolarized imaging (Figure 9C). Metabolic labeling of TRAMP tumors was achieved by injecting either 80 μL of 25% wt/vol [U-^13^C]glucose or 200 μL of 35.73 mg/mL [U-^13^C]glutamine into the tail vein every 15 min for a total labeling time of 45 min [43]. To minimize the effects of stress and anesthesia on metabolism, mice were briefly anesthetized using isoflurane for 2–3 min to perform ^13^C-injection, then allowed to wake between injections. Tissue was collected immediately upon euthanasia and flash-frozen in liquid nitrogen for metabolomic analysis.

For extraction of metabolites, frozen tissue was homogenized using a Tissuelyser LT in 400 μL of cold methanol at 4 °C. Intracellular metabolites were extracted using cold 1:1:1 methanol:water:chloroform [42]. The aqueous fraction was isolated, lyophilized, and resuspended in 400 μL of D_2_O for NMR analysis. For aqueous samples, an external standard of TSP and [2-^13^C]glycine in a 1.5 mm NMR tube was used for quantification.

### 4.3. NMR Acquisition and Quantification

NMR spectra were acquired on an 800 MHz Bruker Avance I (Bruker, Billerica, MA, USA) equipped with a 5 mm triple resonance TXI cryoprobe. High-resolution 1D ^1^H presaturation spectra were acquired with the following parameters: 90° flip angle, 32 k points, 15 ppm sweep width, 12 s water presaturation, 1.4 s acquisition time, 13.4 s repetition time, 64 scans, and 14 min scan time. ^13^C-decoupled ^1^H water presaturation spectra were acquired with the following parameters: 90° flip angle, 24k points, 10 ppm sweep width, 12 s water presaturation, 0.5 s acquisition time, 12.5 s repetition time, 32 scans, and 6 min scan time. Adiabatic decoupling was applied during acquisition using a CHIRP pulse with 54 kHz sweep width (equivalent to 200 ppm @ 800 MHz), and shaped pulse power level of +2 dB with respect to the power level determined for ^13^C-GARP. Phase-sensitive 2D ^1^H-^13^C HSQC was used with the following parameters: 2048 × 4096 points, 6 × 120 ppm sweep width, 1.8 s repetition time, 0.297 s acquisition, 2 averages, J_CH_ = 135 Hz (average J_CH_ of 127, 130, and 145 Hz for glutamate C2, C3, and C4), and 4 h scan time. ^13^C decoupling was not applied during acquisition in order to resolve ^13^C isotopomers. Data were zero-filled in both dimensions to a final digital resolution of 2.9 Hz/point in the F2 dimension. 2D ^1^H-^1^H total correlation spectroscopy (TOCSY) with water presaturation was used with the following parameters: 4096 × 512 points, 12 × 12 ppm sweep width, 2.2 s repetition time, 0.239 s acquisition time, 8 averages, 60 millisecond mixing time, and 2.75 h scan time. Data were zero-filled in both dimensions to a final digital resolution of 9.4 Hz/point in the F2 dimension.

Targeted metabolic profiling was performed using Chenomx (version 8.1, Chenomx Inc, Edmonton, AB, Canada) as previously described [44]. 1D datasets were zero-padded by a factor of 2, apodized with a 0.5 Hz exponential filter, and manually phased and baseline corrected. For cells, intracellular and extracellular metabolite concentrations were quantified from ^1^H water presaturation 1D spectra of unlabeled cell extracts and culture media, respectively. For tissues, total metabolite concentrations were quantified from ^13^C-decoupled ^1^H water presaturation 1D spectra of ^13^C-labeled tissue extracts. As the sequence used for quantification by Chenomx is different from that prescribed, there might be discrepancy in the absolute concentration estimates.

Fractional enrichment (FE) of key metabolites was quantified using a combination of 1D and 2D NMR methods, where FE = [^13^C-labeled metabolite]_HSQC_/[total metabolite]_{13C}1H_. The total metabolite concentration was quantified from ^13^C-decoupled ^1^H spectra by manually fitting peaks of interest using a Lorentzian-Gaussian shape using ACD/1D NMR Processor (version 9) (ACD/Labs, Toronto, ON, Canada). 2D NMR datasets were zero-padded by a factor of 2 and manually phased, and peak volumes were integrated using TopSpin (version 3.5) (Bruker, Billerica, MA, USA). The concentration of ^13^C-labeled metabolites was quantified using ^1^H-^13^C HSQC by integrating the volumes of the cross-peaks and correcting for JCH filtering, inversion efficiency, and number of equivalent protons, using Equation (1), adapted from Heikkinen et al. [45]:

(1) $$Moles_{Met}=\frac{Vol_{Met}}{Vol_{Gly}}\times \frac{N_{1H,Gly}}{N_{1H,Met}}\times \frac{1}{\sin^{2}\left(\frac{\pi J_{CH,Met}}{2J_{CH,Gly}}\right)}\times \frac{{\left(IE_{v,Gly}\right)}^{2}}{{\left(IE_{v,Met}\right)}^{2}}\times Moles_{Gly}$$

where *Vol_Met_* and *Vol_Ref_* are the peak volumes in the HSQC spectrum for the metabolite of interest (*Met*) and the [2-^13^C]glycine (*Gly*), respectively; *N*_1*H,Met*_ and *N*_1*H,Gly*_ are the number of protons for *Met* and [2-^13^C]glycine, respectively; *J_CH,Met_* and *J_CH,Gly_* are the J-coupling in hertz for *Met* and [2-^13^C]glycine; and *IE_ν,Met_* is the inversion efficiency of the pulse at the resonance frequency (ν) of Met. The *J_CH_* coupling constants of key metabolites are provided in Supplementary Table S1. Glutathione FE from [U-^13^C]glutamine labeling studies was quantified using ^1^H-^1^H TOCSY by integrating the peak volumes of the ^13^C-satellites and the central unlabeled peak. A peak table is provided for quantification of metabolites using ^1^H-^13^C HSQC (Supplementary Table S2) and ^1^H-^1^H TOCSY (Supplementary Table S3). The natural abundance ^13^C component (singlet peaks in HSQC) was excluded from the quantification.

### 4.4. ^13^C Isotopomer Modeling

For [U-^13^C]glucose-labeled cell extracts, relative peak volumes of glutamate C3 and glutamate C4 isotopomer multiplets were quantified from high-resolution 2D ^1^H-^13^C HSQC spectra using Topspin (version 4.0.6) (Bruker, Billerica, MA, USA). Glutamate C2 was not included as it has the lowest signal to noise ratio and its doublets (2D12 and 2D23) were not fully resolved. Data were then analyzed using TCACALC (developed by the Advanced Imaging Research Center, University of Texas Southwestern Medical Center) [46]. Model parameters were optimized using nonlinear least squares. Using nomenclature adopted by TCACALC, the parameters examined include relative fluxes through acetyl-CoA synthase (ACS), pyruvate dehydrogenase (PDH), pyruvate carboxylase (YPC), and unidirectional anaplerotic flux of substrates leading to succinyl-CoA relative to citrate synthase (YS). All flux rates are normalized to a citrate synthase (CS) flux of 1, which is equivalent to the TCA cycle flux. Here, the flux through CS is modeled as the sum ACS flux of acetate and other fatty acids and relative ^13^C-glucose flux into the TCA cycle (PDH) (i.e., PDH + ACS = 1). The isotopomer model was validated using ANOVA with the following metrics: (1) statistical fit of individual parameters (*p*-value < 0.05), (2) statistical fit of the overall pathway model [F-value > 100, *p*(F = 0) < 0.0001], and (3) comparison of measured and simulated relative isotopomer areas. The best fit model included the following parameters: ACS, PDH, YS, isotopic enrichment of the ^13^C-tracer, unlabeled “fatty acids” pool to represent all metabolites that convert to acetyl-CoA other than glucose (such as acetate and other ketone bodies), and unlabeled “anaplerotic substrate” to represent all metabolites that enter the TCA cycle through other intermediates of the TCA cycle. For the cells, the absolute TCA flux was calculated as TCA flux = oxygen consumption/(F_c0_ × R_0_ + F_c1_ × R_1_ + F_c2_ × R_2_ + F_c3_ × R_3_ + y × R_a_) [21], accounting for [U-^13^C]glucose as the only carbon-13-enriched substrate provided (R = 3) as well as anaplerotic contribution from unlabeled sources with R_a_ = 1.

### 4.5. Enzyme Activity Assays

To measure lactate dehydrogenase (LDH) activity, cells and tissues were homogenized using a Tissuelyser LT (Qiagen, Germantown, MD, USA) in cell lysis buffer (Cell Signaling Technology, Danvers, MA, USA) and LDH activity was measured spectrophotometrically by quantifying the linear decrease in NADH absorbance at varying pyruvate concentrations at 339 nm using a microplate reader. The maximum velocity (v_max_) and the Michaelis–Menten constant (K_m_) were estimated using the Lineweaver–Burk plot.

To measure PDH activity, cells and tissues were homogenized using a Dounce homogenizer in PBS containing protease inhibitor cocktail (Abcam, Cambridge, UK) and 20 mM NaF to preserve endogenous PDH phosphorylation state and activity. PDH activity was measured spectrophotometrically using the PDH enzyme activity microplate assay kit (Abcam, Cambridge, UK) according to manufacturer’s instructions.

To measure GLS activity, cells and tissues were homogenized using a Tissuelyser LT in lysis buffer provided by the PicoProbe™ glutaminase activity assay kit (Biovision, Milpitas, CA, USA). GLS activity was measured fluorometrically at excite/emission 535 nm/587 nm with a glutamic acid standard curve using a microplate reader.

### 4.6. ATP Quantification

ATP content of cell lysates was measured using the CellTiter-Glo^®^ luminescent cell viability assay kit (Promega, Madison, WI, USA) according to manufacturer’s instructions. Luminescence of the luciferase reaction with ATP was measured using a Veritas luminometer (Thermo Fisher, Waltham, MA, USA).

### 4.7. NAD/NADH and NADP/NADPH Assays

NAD/NADH ratio of cell lysates was measured spectrophotometrically using the NAD/NADH assay kit (Biovision, Milpitas, CA, USA) according to manufacturer’s instructions. Cells were homogenized in lysis buffer provided by the kit using a Tissuelyser LT. NAD, NADH, and their ratio were determined by measuring the reaction of reporter dye with NADH at 450 nm using a microplate reader.

NADP/NADPH ratio of cell lysates was measured spectrophotometrically using the NADP/NADPH assay kit (Abcam, Cambridge, UK) according to manufacturer’s instructions. Cells were homogenized in lysis buffer provided by the kit using a Tissuelyser LT. NADP, NADPH, and their ratio were determined by monitoring NADP formation fluorometrically at excite/emission 540 nm/590 nm using the Tecan Infinite (Tecan Group Ltd., Männedorf, Switzerland) microplate reader.

### 4.8. Glutathione Assay

Reduced glutathione (GSH) and total glutathione of cell lysates were measured spectrophotometrically using the glutathione colorimetric assay kit (Biovision, Milpitas, CA, USA) according to manufacturer’s instructions. Cells were lysed in buffer provided by the kit. GSH, total glutathione, and their ratio were determined by measuring reaction of reporter dye with GSH at 405 nm using a microplate reader.

### 4.9. Oxygen Consumption

Basal oxygen consumption rate (OCR) was measured using a Clark-type O_2_ electrode Oxygraph+ (Hansatech Instruments Ltd., Norfolk, UK). Cells or tissue were placed in a chamber with 1 mL serum-free Dulbecco’s Modified Eagle’s Medium (DMEM), continuously mixed at 37 °C, and respiration was assessed over 1 min. The medium used in the chamber (DMEM standard formulation with 25 mM glucose and 5 mM glutamine) for the oxygen measurements was similar to that used for isotopic labeling (RPMI with 25 mM glucose and 10 mM glutamine with either substrate uniformly carbon-13-labeled). Control traces using medium alone were acquired after each sample to assess oxygen consumption attributed to the electrochemistry of the Clark electrode.

### 4.10. Seahorse Analysis

OCR was measured using a Seahorse XFe24 Extracellular Flux Analyzer (Agilent, Santa Clara, CA, USA). Cells were seeded in Seahorse XFe24 microplates at ~60,000 cells/well and incubated overnight. Medium was then replaced with Seahorse XF Assay Medium, and cells were incubated in a CO_2_-free incubator at 37 °C for 1 h prior to loading into the XFe24. The measurement protocol consisted of 3 min mix, 2 min wait, and 3 min measurement cycles at 37 °C, allowing for OCR measurements every 8 min. OCR measurements were normalized to protein concentration using the Bradford Assay. For the Mito Fuel Flex Kit, optimal concentrations of BPTES (3 μM), etomoxir (4 μM), and UK5099 (2 μM) were used.

### 4.11. HP ^13^C NMR Spectroscopy of Cell Slurries

Approximately 24 μL of 14.2 M [1-^13^C]pyruvate with 15 mM trityl radical (GE Healthcare, Chicago, IL, USA) was polarized in a HyperSense polarizer (Oxford Instruments, Abingdon, UK) for 1 h followed by rapid dissolution in a superheated isotonic buffer (4.5 mL) with 0.3 mM EDTA and equivalents of NaOH (80 mM) to yield a pH of 6.5–8. Each cell slurry of 10–40 million cells resuspended in 200 μL of serum-free DMEM medium was kept on ice and allowed to equilibrate to 37 °C at the start of dissolution, then injected with equivalent volume of 80 mM HP [1-^13^C]pyruvate. Cell count did not affect results as 10 million cells were sufficient to fill the sensitive region of the coil. Dynamic ^13^C NMR spectra were acquired every 4 s using 10° pulses at 37 °C using a 60 MHz benchtop NMR spectrometer (Oxford Instruments, Abingdon, UK) equipped with a 5-mm ^1^H/^13^C probe. The spectra were zero-filled by a factor of 2, Fourier-transformed, phased, and baseline-corrected. Pyruvate and lactate peaks at each time point were manually fitted with a Lorentzian–Gaussian shape. The lactate to pyruvate ratio area under the entire dynamic curve was computed.

### 4.12. TRAMP Hyperpolarized ^13^C MRI

Hyperpolarized MR imaging of TRAMP mice was performed on a GE 3T human scanner with a custom-designed, dual-tuned mouse coil. However, to ensure adequate size of prostate tumors prior to hyperpolarized studies, mice were imaged on the 14T vertical bore Varian microimaging imaging system (Agilent Technologies, Santa Clara, CA, USA). All studies were performed under the aegis of an animal protocol approved by the UCSF Institutional Animal Care and Use Committee. T_2_-weighted ^1^H anatomical MR images were acquired using a fast spi-echo (FSE) sequence in sagittal, axial, and coronal views. Approximately 24 μL of 14.2 M [1-^13^C]pyruvate with 15 mM trityl radical (GE Healthcare, Chicago, USA) was polarized on a Hypersense polarizer (Oxford Instruments, Abingdon, UK) for 1 h as previously described [10] followed by dissolution in 4.5 mL 50 mM phosphate buffer with 0.3 mM EDTA and equivalents of NaOH (80 mM) to obtain physiological temperature and pH. Approximately 300 μL of hyperpolarized solution was injected via a pre-established jugular or tail vein catheter over 12 s followed by a chase of 150 μL of heparinized saline. A 3D CSI sequence was initiated at 35 s after injection and HP-^13^C signal acquired over 14 s. A double spin-echo pulse sequence with small tip-angle excitation, adiabatic refocusing and flyback echo-planar readout trajectory [10], and an 8 × 8 × 16 matrix and 40 × 40 × 86.4 mm field-of-view (0.135 cc resolution) was used to acquire in vivo 3D hyperpolarized ^13^C MRSI data in 14 s after the animals were injected with hyperpolarized [1-^13^C]pyruvate. After baseline imaging, the mice underwent orchiectomy and the hyperpolarized imaging was repeated again ~5 days (±2 days) later. Mice were categorized into ADPC and CRPC as detailed above following the modified RECIST criteria.

### 4.13. Statistics

Student’s *t*-test was used to compare human and murine ADPC and CRPC models using Prism (GraphPad) version 8.3. All statistics are reported as mean ± standard error. *p* values less than 0.05 (* *p* < 0.05, ** *p* < 0.01 and *** *p* < 0.001) were considered significant.

## 5. Conclusions

In summary, our findings using human and murine models are consistent with the hypothesis that castration-resistant growth alters several metabolic pathways, including glycolysis, glutaminolysis, TCA metabolism, and glutathione redox capacity. These pathways may provide biomarkers to predict the development of clinical CRPC or candidate drug targets for the treatment of CRPC in patients.

## Data Availability

The data presented in this study are available in this article and supplementary material.

## Supplementary Materials

The following are available online at https://www.mdpi.com/2218-1989/11/3/139/s1, Figure S1: Conventional 1D ^1^H presaturation spectra of LNCaP (blue) and PC-3 (red) cells labeled with A, [U-^13^C]glucose and B, [U-^13^C]glutamine. Zoomed region indicates glutamate C4 region with vertical scale increased by 2-fold. 2D ^1^H-^13^C HSQC of LNCaP and PC-3 cell extracts labeled with C, [U-^13^C]glucose and D, [U-^13^C]glutamine. E, 2D ^1^H-^1^H TOCSY of [U-^13^C]glutamine-labeled extracts of LNCaP and PC-3 show peaks associated with glutamate (grey), glutamine (pink), and glutathione (green). Dotted lines indicate ^13^C-satellites. Arrows indicate unenriched metabolite peaks. Metabolite peaks are labeled as follows: 1. ^13^C-Lactate, 2. Lactate, 3. ^13^C-Alanine, 4. Alanine, 5. ^13^C-Glutamate, 6. Glutamate, 7. ^13^C-Glutamine, 8. Glutamine, 9. ^13^C-Glutathione, 10. ^13^C-Aspartate, 11. ^13^C-Malate, 12. ^13^C-Citrate, 13. Glutathione, Figure S2: Representative 1D ^1^H presaturation with (pink) and without (green) ^13^C-decoupling in PC-3 cells labeled with A, [U-^13^C]glucose and B, [U-^13^C]glutamine. Metabolite peaks are labeled as follows: 1. ^13^C-Lactate, 2. Lactate, 3. ^13^C-Alanine, 4. Alanine, 5. ^13^C-Glutamate, 6. Glutamate, 7. ^13^C-Glutamine, 8. Glutamine, 9. ^13^C-Glutathione, 10. Glutathione, Figure S3: TRAMP tumor changes with onset of castration-resistance. A, Changes in tumor volume (cc) in ADPC and CRPC TRAMP mice from baseline to 5 days (±1 day) post castration. B, Lac/Pyr ratio of the TRAMP tumors at baseline depict no significant difference between the ADPC and CRPC tumors. C, Bar graph showing the differential staining of Ki67 between the ADPC and CRPC TRAMP tumors. Figure S4. Change in volume of ADPC and CRPC TRAMP tumors one-week post-orchiectomy used for glucose and glutamine labeling studies. Table S1: Steady-state metabolite concentrations of unlabeled LNCaP and PC-3 cell extracts (nmol/million cells, *n* = 4) and androgen-dependent and castration-resistant TRAMP tumors (nmol/mg wet tissue, *n* = 3), Table S2: 2D ^1^H-^13^C HSQC chemical shifts and J_CH_ constants used for quantifying ^13^C-labeled metabolites, Table S3: 2D ^1^H-^1^H TOCSY chemical shifts of unlabeled metabolite peaks and ^13^C-satellites used for quantifying extracts labeled with [U-^13^C]glutamine.
