# Supplementary material for: Resistance to Androgen Deprivation Leads to Altered Metabolism in Human and Murine Prostate Cancer Cell and Tumor Models
Source: Metabolites. 2021 Feb 26;11(3):139. doi: 10.3390/metabo11030139 (PMC7996870; doi:10.3390/metabo11030139)
Supplement: Supplementary file 1 [file metabolites-11-00139-s001.pdf]

Supplemental Information

Resistance to Androgen Deprivation Leads to Altered Metabolism in Human and Murine Prostate Cancer Cell and Tumor Models

Jinny Sun[1], Robert Bok[2], Justin Delos Santos[2], Deepti Upadhyay[2], Romelyn Delos Santos[2], Shubhangi Agarwal[2], Mark Van Criekinge[2], Daniel B. Vigneron[2], Rahul Aggarwal[3], Donna M. Peehl[2], John Kurhanewicz[2*], and Renuka Sriram[2*]

[1] Graduate Program in Bioengineering, University of California, Berkeley and University of California, San Francisco

[2] Department of Radiology and Biomedical Imaging, University of California, San Francisco

[3] Hematology/Oncology, University of California, San Francisco

* Correspondence: renuka.sriram@ucsf.edu (R.S.); john.kurhanewicz@ucsf.edu (J.K.)

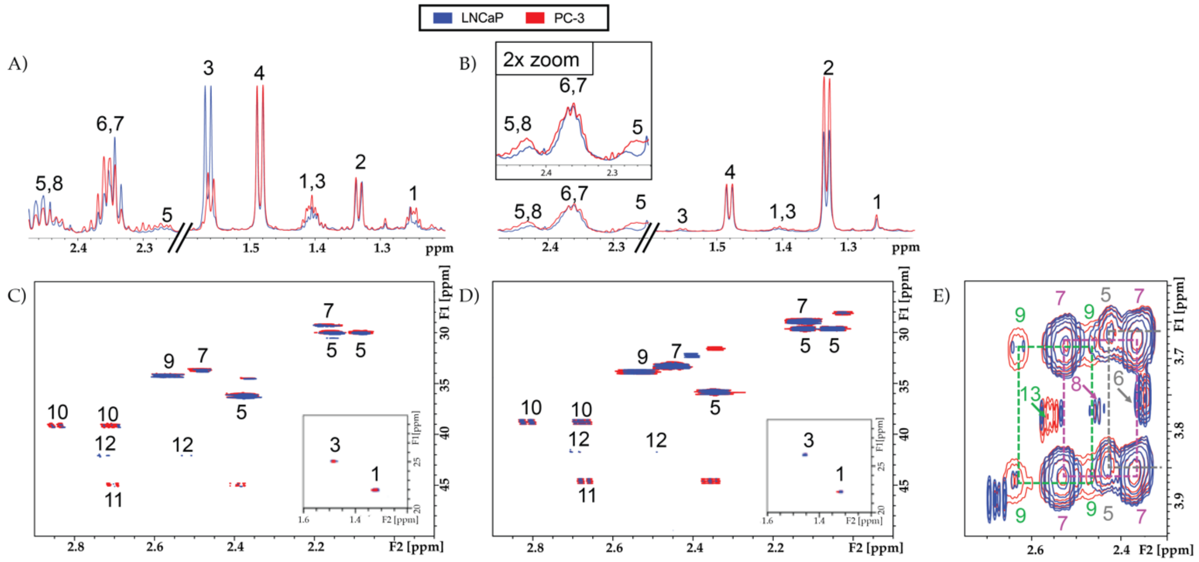

Figure S1. Conventional 1D ^{1}H presaturation spectra of LNCaP (blue) and PC-3 (red) cells labeled with **A,** [U-^{13}C]glucose and **B,** [U-^{13}C]glutamine. Zoomed region indicates glutamate C4 region with vertical scale increased by 2-fold. 2D ^{1}H-^{13}C HSQC of LNCaP and PC-3 cell extracts labeled with **C,** [U-^{13}C]glucose and **D,** [U-^{13}C]glutamine. **E,** 2D ^{1}H-^{1}H TOCSY of [U-^{13}C]glutamine-labeled extracts of LNCaP and PC-3 show peaks associated with glutamate (grey), glutamine (pink), and glutathione (green). Dotted lines indicate ^{13}C-satellites. Arrows indicate unenriched metabolite peaks. Metabolite peaks are labeled as follows: 1. ^{13}C-Lactate, 2. Lactate, 3. ^{13}C-Alanine, 4. Alanine, 5. ^{13}C-Glutamate, 6. Glutamate, 7. ^{13}C-Glutamine, 8. Glutamine, 9. ^{13}C-Glutathione, 10. ^{13}C-Aspartate, 11. ^{13}C-Malate, 12. ^{13}C-Citrate, 13. Glutathione.

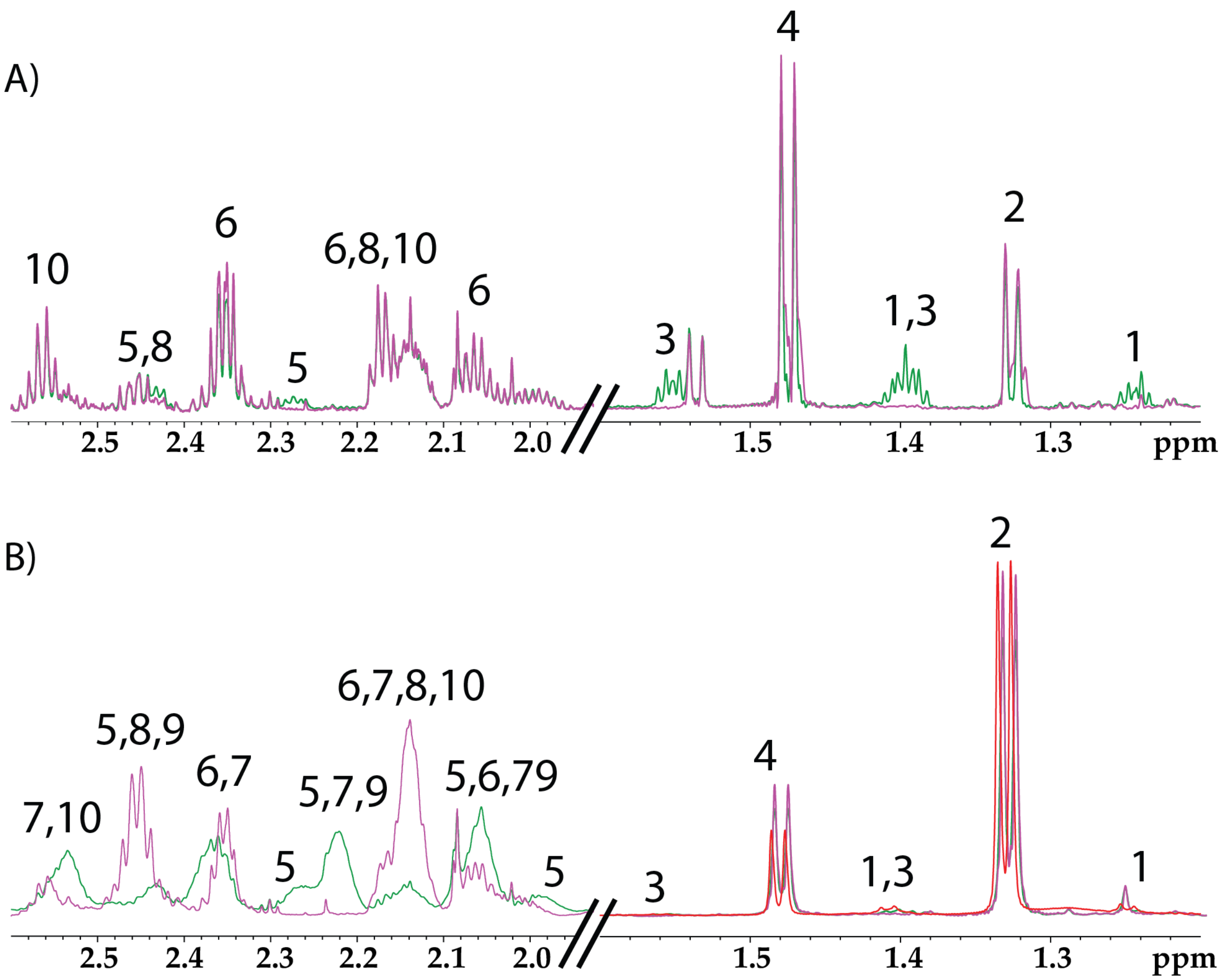

Figure S2. Representative 1D ^{1}H presaturation with (pink) and without (green) ^{13}C-decoupling in PC-3 cells labeled with **A,** [U-^{13}C]glucose and **B,** [U-^{13}C]glutamine. Metabolite peaks are labeled as follows: 1. ^{13}C-Lactate, 2. Lactate, 3. ^{13}C-Alanine, 4. Alanine, 5. ^{13}C-Glutamate, 6. Glutamate, 7. ^{13}C-Glutamine, 8. Glutamine, 9. ^{13}C-Glutathione, 10. Glutathione.

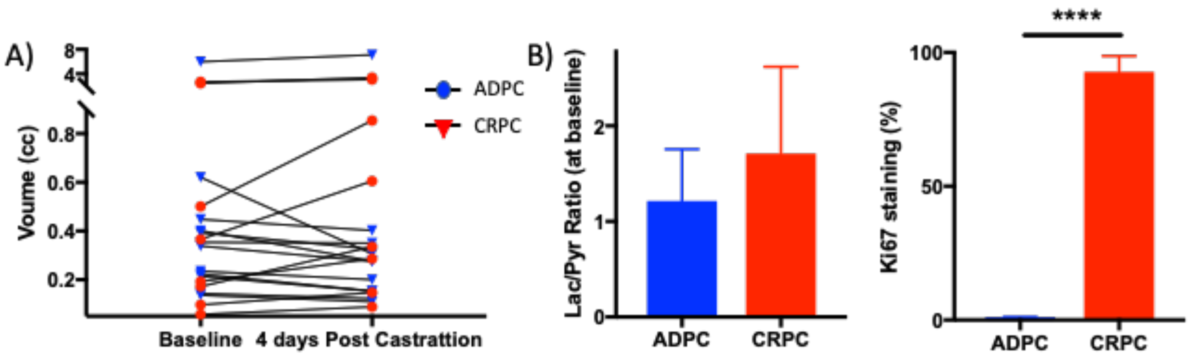

Figure S3. TRAMP tumor changes with onset of castration-resistance**. A**, Changes in tumor volume (cc) in ADPC and CRPC TRAMP mice from baseline to 5 days (± 1 day) post castration. **B**, Lac/Pyr ratio of the TRMAP tumors at baseline depict no significant difference between the ADPC and CRPC tumors. **C**, Bar graph showing the differential staining of Ki67 between the ADPC and CRPC TRAMP tumors.

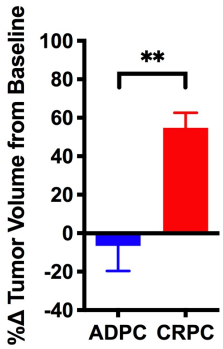

Figure S4. Change in volume of ADPC and CRPC TRAMP tumors one-week post-orchiectomy used for glucose and glutamine labeling studies.

Table S1. Steady-state metabolite concentrations of unlabeled LNCaP and PC-3 cell extracts (nmol/million cells, N=4) and androgen-dependent and castration-resistant TRAMP tumors (nmol/mg wet tissue, N=3).

	LNCaP	PC-3		ADPC TRAMP	CRPC TRAMP	
Metabolite	Average ± SE	Average ± SE	Raw p-value	Average ± SE	Average ± SE	Raw p-value
Acetate	7.6 ± 1.2	4.1 ± 1.4	0.1167	5.6 ± 3.7	2.6 ± 0.6	0.4666
Alanine	24.5 ± 1.9	29.6 ± 0.3	**0.0366**	12.8 ± 7.1	16.2 ± 1.3	0.6587
Aspartate	18.9 ± 1.3	31.5 ± 0.5	**0.0001**	7.2 ± 2.0	4.9 ± 0.3	0.3377
Choline	2.2 ± 0.3	2.3 ± 0.1	0.7081	1.6 ± 0.1	2.5 ± 1.7	0.6141
Citrate	22.3 ± 1.0	3.5 ± 0.2	**<0.0001**	2.6 ± 0.2	1.4 ± 0.3	**0.0268**
Creatine	23.0 ± 1.0	5.2 ± 0.2	**<0.0001**	20.7 ± 1.2	11.3 ± 2.2	**0.0198**
Creatine phosphate	31.5 ± 1.9	7.3 ± 0.4	**<0.0001**	1.9 ± 0.7	1.0 ± 0.3	0.2664
Glucose	19.6 ± 1.1	10.5 ± 1.3	**0.0022**	21.1 ± 1.1	10.9 ± 4.0	0.0714
Glutamate	62.3 ± 3.0	82.2 ± 1.0	**0.0008**	26.0 ± 11.6	21.1 ± 1.4	0.6936
Glutamine	65.1 ± 2.9	60.6 ± 3.7	0.3761	7.3 ± 3.3	5.2 ± 0.6	0.5800
Glutathione	16.2 ± 1.2	30.2 ± 4.0	**0.0155**	5.0 ± 0.5	6.8 ± 0.7	0.0969
Glycerophosphocholine	17.8 ± 1.5	23.9 ± 1.6	**0.0308**	11.9 ± 2.9	13.2 ± 4.1	0.8028
Lactate	40.8 ± 2.0	60.7 ± 1.6	**0.0017**	30.7 ± 9.3	77.8 ± 2.0	**0.0078**
myo-Inositol	11.7 ± 1.9	84.8 ± 1.1	**<0.0001**	14.7 ± 5.4	3.7 ± 0.3	0.1120
Phosphocholine	40.8 ± 2.0	60.7 ± 1.6	**0.0002**	13.3 ± 2.7	10.2 ± 2.3	0.4379
Pyruvate	3.1 ± 0.1	2.3 ± 0.4	0.0763	n.d.	n.d.	–
Succinate	1.0 ± 0.1	1.6 ± 0.3	0.1088	n.d.	n.d.	–
Threonine	7.8 ± 0.4	6.9 ± 2.1	0.6936	n.d.	n.d.	–
Total Choline	60.8 ± 3.2	86.9 ± 3.0	**0.0010**	26.8 ± 5.5	26.0 ± 7.8	0.9356

*Total choline was defined as the summed concentrations of choline, phosphocholine, and glycerophosphocholine.

Table S2. 2D ^{1}H-^{13}C HSQC chemical shifts and J_{CH} constants used for quantifying ^{13}C-labeled metabolites

F2 (ppm)	F1 (ppm)	Compound	J_{CH} (Hz)
0	0	TSP	
1.31	22.9	Lactate C3	128 [1]
1.49	19.02	Alanine C3	130 [1]
2.08	29.82	Glutamate C3	130 [1,2]
2.12	29.29	Glutamine C3	131 [2]
2.15	29.06	Glutathione C7 (Glutamate C3)	132
2.34	36.36	Glutamate C4	127 [1,2]
2.34	45.46	Malate C3	
2.44	33.92	Glutamine C4	128 [2]
2.52	48.71	Citrate C2,4	128
2.55	34.05	Glutathione C6 (Glutamate C4)	130
2.65	45.46	Malate C3	
2.66	48.71	Citrate C2,4	128
2.71	39.33	Aspartate C3	129 [1,2]
2.8	39.48	Aspartate C3	130 [1,2]
3.23	76.96	Glucose C2b	144 [3,4]
3.39	72.34	Glucose C2a	144 [3,4]
3.46	78.57	Glucose C3,5b	144 [3,4]
3.52	74.2	Glucose C2,4a	144 [3,4]
3.55	44.3	Glycine C2	143 [1]
3.7	75.64	Glucose C3a	143 [3], 145 [4]
3.74	63.35	Glucose C6	144 [3]
3.74	63.35	Glucose C6	144 [3]
3.74	63.35	Glucose C6	144 [3]
3.74	57.64	Glutamate C2	145 [2]
3.78	57.23	Glutamine C2	145 [2]
3.77	53.56	Alanine C2	–
3.77	57.17	Glutathione C8 (Glutamate C2)	143.5
3.82	74.14	Glucose C2,4a	144 [3, 4]
3.83	63.41	Glucose C6	144 [3]
3.83	63.41	Glucose C6	144 [3]
3.9	63.47	Glucose C6	144 [3]
3.91	55.08	Aspartate C2	144 [2]

4.1	71.37	Lactate C2	–
4.29	73.24	Malate C2	–
4.63	98.71	Glucose C1b	162 [4]
5.22	94.93	Glucose C1a	169 [4]

Table S3. 2D ^{1}H-^{1}H TOCSY chemical shifts of unlabeled metabolite peaks and ^{13}C-satellites used for quantifying extracts labeled with [U-^{13}C]glutamine

Compound	^{1}H-^{12}C (F2, F1) Main peak	^{1}H-^{13}C (F2, F1) Satellite peaks
Glutamate C4	(2.34, 3.75)	(2.27, 3.67), (2.27, 3.85), (2.42, 3.66), (2.42, 3.85)
Glutamine C4	(2.45, 3.78)	(2.37, 3.69), (2.37, 3.87), (2.53, 2.69), (2.53, 3.87)
Glutathione C3	(2.54, 3.78)	(2.47, 3.70), (2.47, 3.88), (2.62, 3.70), (2.62, 3.88)

Table S4. Correction factor for absolute concentrations of metabolites (+ indicates over-estimation and – indicates under-estimation of the metabolite concentrations).

**Metabolites**	**% Error**
Glucose	-10.7
Lactate	+17.5
Alanine	+7.2
Glutamate	-21.8
Glutamine	-7.7
Aspartate	19.2
Choline	-1.5
Phosphocholine	-23.8
GPC	-20.8

References

1. Levin YS, Albers MJ, Butler TN, Spielman D, Peehl DM, Kurhanewicz J. Methods for metabolic evaluation of prostate cancer cells using proton and 13C HR-MAS spectroscopy and [3- 13C] pyruvate as a metabolic substrate. Magn Reson Med. 2009;62:1091–8.
2. Deelchand DK, Uğurbil K, Henry PG. Investigating brain metabolism at high fields using localized 13C NMR spectroscopy without 1H decoupling. Magn Reson Med. Wiley Subscription Services, Inc., A Wiley Company; 2006;55:279–86.
3. Tiainen M, Maaheimo H, Soininen P, Laatikainen R. 13C isotope effects on 1H chemical shifts: NMR spectral analysis of 13C-labelled D-glucose and some 13C-labelled amino acids. Magnetic Resonance in Chemistry. John Wiley & Sons, Ltd; 2010;48:117–22.
4. Yu B, van Ingen H, Vivekanandan S, Rademacher C, Norris SE, Freedberg DI. More accurate 1JCH coupling measurement in the presence of 3JHH strong coupling in natural abundance. Journal of Magnetic Resonance. Academic Press; 2012;215:10–22.
